# Study protocol for “In-vehicle sensors to detect changes in cognition of older drivers”

**DOI:** 10.1186/s12877-023-04550-5

**Published:** 2023-12-14

**Authors:** Ruth Tappen, David Newman, Monica Rosselli, Jinwoo Jang, Borko Furht, KwangSoo Yang, Seyedeh Gol Ara Ghoreishi, Jiannan Zhai, Joshua Conniff, Muhammad Tanveer Jan, Sonia Moshfeghi, Somi Panday, Kelley Jackson, Marie Adonis-Rizzo

**Affiliations:** 1 Christine E. Lynn College of Nursing, Florida Atlantic University, 777 Glades Road, Boca Raton, FL 33431 USA; 2 Department of Psychology, Florida Atlantic University, 3200 College Ave, Davie, FL 33314 USA; 3 Department of Civil, Environmental, and Geomatics Engineering, Florida Atlantic University, 777 Glades Road, Boca Raton, FL 33431 USA; 4 Department of Electrical Engineering and Computer Science, Florida Atlantic University, 777 Glades Road, Boca Raton, FL 33431 USA; 5 Florida Atlantic University, 777 Glades Road, Boca Raton, FL 33431 USA; 6 I-SENSE, Florida Atlantic University, 777 Glades Road, Boca Raton, FL 33431 USA; 7 Neuropsychology Lab, Florida Atlantic University, 777 Glades Road, Boca Raton, FL 33431 USA

**Keywords:** Driving, Cognitive change, Video, Telematics, Mild cognitive impairment, Older driver, Sensors

## Abstract

**Background:**

Driving is a complex behavior that may be affected by early changes in the cognition of older individuals. Early changes in driving behavior may include driving more slowly, making fewer and shorter trips, and errors related to inadequate anticipation of situations. Sensor systems installed in older drivers’ vehicles may detect these changes and may generate early warnings of possible changes in cognition.

**Method:**

A naturalistic longitudinal design is employed to obtain continuous information on driving behavior that will be compared with the results of extensive cognitive testing conducted every 3 months for 3 years. A driver facing camera, forward facing camera, and telematics unit are installed in the vehicle and data downloaded every 3 months when the cognitive tests are administered.

**Results:**

Data processing and analysis will proceed through a series of steps including data normalization, adding information on external factors (weather, traffic conditions), and identifying critical features (variables). Traditional prediction modeling results will be compared with Recurring Neural Network (RNN) approach to produce Driver Behavior Indices (DBIs), and algorithms to classify drivers within age, gender, ethnic group membership, and other potential group characteristics.

**Conclusion:**

It is well established that individuals with progressive dementias are eventually unable to drive safely, yet many remain unaware of their cognitive decrements. Current screening and evaluation services can test only a small number of individuals with cognitive concerns, missing many who need to know if they require treatment. Given the increasing number of sensors being installed in passenger vehicles and pick-up trucks and their increasing acceptability, reconfigured in-vehicle sensing systems could provide widespread, low-cost early warnings of cognitive decline to the large number of older drivers on the road in the U.S. The proposed testing and evaluation of a readily and rapidly available, unobtrusive in-vehicle sensing system could provide the first step toward future widespread, low-cost early warnings of cognitive change for this large number of older drivers in the U.S. and elsewhere.

## Background

About 1 in 9 (10.7%) people in the US age 65 and older has Alzheimer’s disease or related dementia; another 15 to 20% have mild cognitive impairment (MCI), and one-third of these will develop dementia within 5 years [1]. Older African Americans and Hispanics have a higher prevalence rate, probably related to higher rates of diabetes, cardiovascular disease, and the effects of socioeconomic disadvantage [2], reinforcing the importance of culturally diverse samples in Alzheimer’s research. Individuals with dementia eventually cannot perform complex everyday activities, including driving. Interestingly, the neuropathologies of AD (Alzheimer’s Disease) have been found in the brains of older drivers killed in motor vehicle accidents who did not even know they had AD and had no apparent signs of it [3].

Older drivers may be at greater risk of collision in the years before AD diagnosis [4]. One analysis identified a five-fold increase in crashes 3 years before the diagnosis of dementia and a higher likelihood of failing a road test (risk ratio = 10.77) [5]. Similarly, Fraade‐Blanar and colleagues [6] found that a one unit-lower Cognitive Abilities Screening Instrument scored using item response theory (CAS-IRT) predicted a crash incidence rate of 1.25 in a sample of 2,165 older group health enrollees. Drivers with dementia make twice as many mistakes as controls and fail on-the-road tests at a rate of 67% compared to 3% for controls [7]. Pavlou and colleagues [8] reported that driving behavior changes such as speed, lateral position, reaction time, following distance, left turns, and time off the road distinguish normal controls from those with cerebral pathologies.

Previous research has focused on older drivers with dementia, but interest in the driving behavior of individuals with MCI is increasing. Few studies have reported the use of continuous, unobtrusive sensors and related monitoring devices for detecting subtle variability in the performance of highly complex everyday activities over time. Eby and colleagues [9] employed in-vehicle technology to monitor driving performance for 2 months. Compared with 26 unimpaired drivers, 17 drivers with early-stage dementia were found to have significantly restricted driving space and were more likely to get lost, even though they had been cleared for driving. In a small sample of 21 unimpaired older adults and 7 with MCI who were followed for 200 days using a sensing device, those with MCI drove fewer miles and fewer of these miles on highways [10].

The Long Road study (2021) is among the first longitudinal studies showing the usefulness of naturalistic data and machine learning techniques to detect MCI and dementia from driving behavior [11]. Among 2977 older drivers studied, 64 were identified as having MCI or dementia through a review of their medical records and annual interview. Age was predictive of MCI and dementia, followed by the percentage of trips traveled within 15 miles of home, race/ethnicity, minutes per trip chain (i.e., length of trips starting and ending at home), minutes per trip, and the number of challenging braking events with deceleration rates 0.35 g. The point of conversion to MCI or dementia could not be determined, at least partly due to the data collection method. Data from our naturalistic, longitudinal study may contribute to identifying these time points.

Similarly, Bayat and colleagues [12] conducted a study using machine learning methods to evaluate the ability of in-vehicle GPS to distinguish drivers with preclinical AD from those without preclinical AD. Using four Random Forest (RF) models with three sets of variables, driving features only, driving features and age, and driving features, age, andAPOE*ε*4 status, it was found that prediction of preclinical AD was 82% using GPS-based driving indicators, 88% using age and driving indicators, and 91% using age, APOE *ε*4 status, and driving.

Davis et al. [13] performed a pilot study using in-vehicle technology (GPS, video) to capture driving behaviors and errors of adult drivers with preclinical AD and early symptomatic AD compared to cognitively normal adults. In early AD, g-force (a vector of acceleration) events produced common errors predominately related to inadequate anticipation of situations, such as late response or driving too fast, mistakes of judgment, and frequent traffic violations. Davis et al. [13] suggest that an event-based approach rather than costly continuous video monitoring to assess driving risk behaviors can be more efficient. Those with preclinical AD drove more slowly and had the lowest number of aggressive events over 3 months. An important limitation of the study was that cognitively intact individuals (CDR = 0) did not have video installed in their vehicles.

The rationale for the current research arises from the importance of identifying cognitive dysfunction as early and efficiently as possible. An estimated 4 to 8 million older adults with MCI are currently driving [14]. This significant proportion of older drivers constitutes a previously unexplored opportunity to detect cognitive decline. In this study, we will systematically examine how current in-vehicle technologies may detect anomalous driving behavior indicative of cognitive impairment.

## Methods/design

### Objective

The objective of this study is to test an unobtrusive multi-sensor system’s ability to detect cognitive change in older (≥ 65) drivers.

### Aims

To achieve this goal, the In-Vehicle study has the following aims:

1. Test the ability of a package of in-vehicle sensors to detect cognitive change:
  - Establish baseline cognitive status and driving behaviors.
  - Examine sensor data output for the ability to detect the cognitive change in individual measures, latent constructs, and diagnostic groupings (e.g., change from unimpaired to MCI) over time.
  - Identify cognitive changes that in-vehicle sensors can detect.
  - Identify the sensor system components that best predict these significant changes in cognition.
2. Develop algorithms that can:
  - Translate sensor data into Driving Behavior Indices (DBIs) that are gender, age, and vehicle specific.
  - Examine the accuracy and stability of the DBIs by comparing DBIs generated using exponential smoothing and ARIMA techniques to DBIs that are adjusted for weather and road conditions derived from national and state databases.
  - Provide interpretable indicators of change in DBI and associated decline in the underlying cognitive functions.
3. Evaluate the acceptability to older drivers of the installed systems.
  - Evaluate participants’ response to the sensor system in terms of their awareness of the installed system, obtrusiveness, driver distraction, reported effect on driving behavior, and overall acceptability.

### Study design

This study will utilize a naturalistic longitudinal parallel mixed methods design. The quantitative phase of this research will be a longitudinal forecasting design with latent constructs. The qualitative phase will use content analysis to investigate the drivers’ perception of acceptability and the unobtrusive nature of the sensor unit.

### Sample size calculation

The average national percent change in cognition disaggregated by age groups (65–74, 75–84, 85+) over a 3-year period [15] was used to calculate number of participants. G*Power 3.1.9.2. was used to calculate the total number of participants predicted to show measurable change in cognition required to obtain power = 0.80 with an alpha = 0.05, 2 groups (Change, No-Change), 12 repeated measures, a correlation between measures of 0.5 and a medium effect size (Cohen’s f = 0.0.17) which is 200 using the repeated measures algorithm in G*3.1.9.2. With an average of 52.1% of the participants estimated to show a minimum change over the 3-year period, a total sample of 384 participants is required. Due to their age and driving requirements of the participants in this study, a 20% attrition rate [16] was used to calculate the total number of participants to be enrolled in this study. This resulted in a total required sample of 460.

### Study eligibility

#### Inclusion criteria

Age 65 and older, a valid driver’s license, evidence of insurance, use of a passenger car or pickup truck, age and education-adjusted Montreal Cognitive Assessment (MoCA) [17] score of 19 or higher, and a willingness to return to one of three testing/installation sites for retesting, data download, and sensor maintenance as needed every 3 months, are all requirements for inclusion. Potential participants must also be able to speak English, Spanish, or Haitian Creole and pass the standard state driver’s license requirements for vision, hearing, strength, and flexibility.

#### Exclusion criteria

Those who are under 65, do not have a vehicle to drive, a valid driver’s license or insurance, score below 19 on the MoCA [17], cannot return every 3 months for retesting and sensor maintenance, cannot pass the physical screening, or decline to sign the Institutional Review Board-approved written consent, or are not fluent in either Spanish, English or Creole, will be excluded. We will exclude those with a clinically significant active illness, neurological or psychiatric disorders, or loss of consciousness within the last 5 years and those who cannot meet the state physical ability requirements for vision or hearing.

### Participant recruitment

A community outreach approach to participant recruitment will be led by a dedicated study recruiter with prior research experience. The recruitment effort will begin with networking with representatives of community-based senior services, either at networking events or in individual meetings. This high-level networking is directed to developing connections with organizations that serve the age 55+ segment of the population to elicit invitations to conduct formal and informal information sessions with the older clients/members/residents in their communities, programs, and services. This includes senior living communities, senior day programs, places of worship, and, secondarily, health services. Presentations and discussions with potential participants will be done in English, Spanish, and Haitian Creole. Bilingual research team members will join the study recruiter to conduct the meetings with primarily non-English speaking groups. Printed materials about the study will be available in these languages, as well as information on how to connect with the study team to enroll. Another smaller recruiting effort will be primarily participant-driven. With Institutional Review Board approval, participants who introduce an eligible individual to the study are given an additional gift card for each new enrollee referred.

Individuals who express interest in participation receive further explanation of the study and what participation entails. They are then briefly screened for eligibility, and an appointment is made. On the first visit, further explanation of the study is provided, consent is obtained, eligibility is confirmed, and baseline tests are administered. A sensor installation team places the telematic and video sensors in the participants’ vehicle once consent is obtained and eligibility confirmed.

### Sensor instrumentation

The in-vehicle sensor network uses open-source hardware and software components to reduce the time, risks, and costs associated with developing in-vehicle sensing units. In-vehicle sensor systems are kept simple and compact by minimizing complex wiring, limiting the size of the sensing units, and limiting the number of sensors in a vehicle to support the unobtrusiveness of in-vehicle sensors. Each in-vehicle sensor system is comprised of two distributed sensing units: one for telematics data and the other for video data.

#### Telematic units

The telematics unit of the in-vehicle sensor system is built upon Raspberry Pi which enables the modification and adaptation of telematics sensor data types, sampling frequencies, and onboard data logging algorithms. A vehicle’s On-Board Diagnostic (OBD) port provides 12-V power to a telematics unit. Furthermore, the telematics unit has a smart power control system based on the voltage of an OBD port to prevent battery drainage. The telematics unit uses ten mA (milliamps) up to 500 mA when a vehicle is in use or not in use, respectively. The processor of the telematics units has a heat sink to provide stable and reliable operations in Florida’s hot climate.

The telematics sensor provides three data types: Inertial measurement unit (IMU), OBD, and GPS data. IMU data comprise 3-axial acceleration and 3-axial gyroscope (e.g., angular velocity) to capture vehicles’ dynamic motions and orientations. IMU data will be processed to determine hard braking, hard accelerations and hard turns, and GPS data. It also includes a timestamp, latitude, longitude, altitude, course over ground (COG), and the number of communicating satellites. The first four will constitute the trajectory of a vehicle and be used to analyze travel patterns (e.g., travel distance, trip purpose). COG data will be used to calculate the heading information of a vehicle. The number of satellites connected to GPS sensors will be used to analyze the accuracy of GPS information. A minimum of four satellites provides the roughest estimation of location, while 7 to 8 satellites are needed to estimate an accurate location within 10–11 yards [18]. The sampling rates of the IMU sensor, gyroscope, and GPS data are set to 12.5 Hz, 25 Hz, and 1 Hz, respectively.

**Fig. 1:**
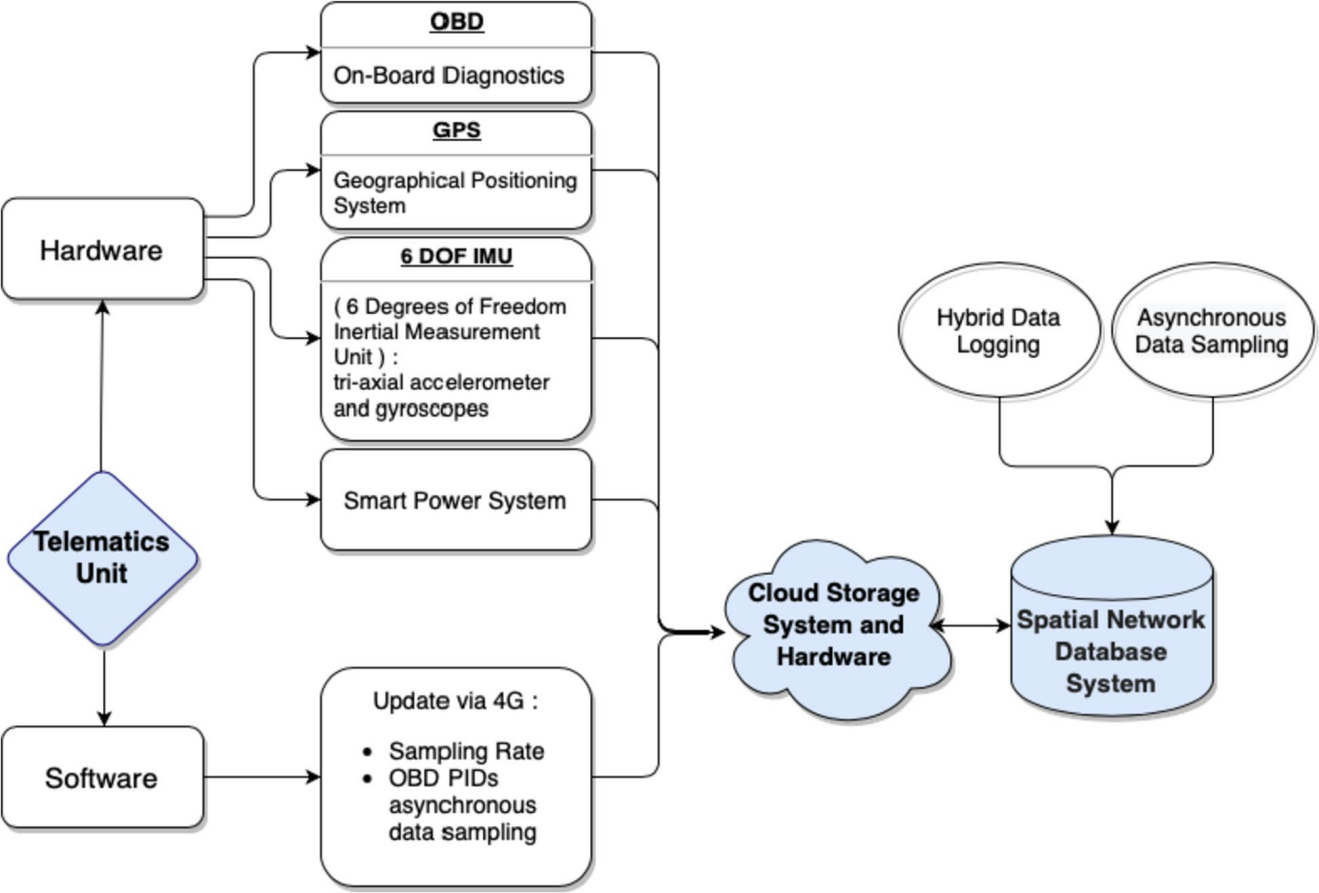
The architecture of telematic units

The telematics sensor employs hybrid data logging systems: onboard and cloud data logging. High-resolution telematics data will be stored in local (in vehicle) data stores, manually collected and uploaded to a secure central database during participants’ quarterly visits. The in-vehicle sensor has cellular connectivity. The Cloud database will be used primarily to check the operating state of in-vehicle sensors (e.g., error messages, local data connectivity, operating condition of camera units through Wi-Fi connections), software updates through 4G connections (e.g., change sampling rates, update OBD parameters, firmware update), and troubleshoot malfunctioning devices (e.g., remote control through 4G connections). See Fig. 1.

#### Video unit

**Fig. 2:**
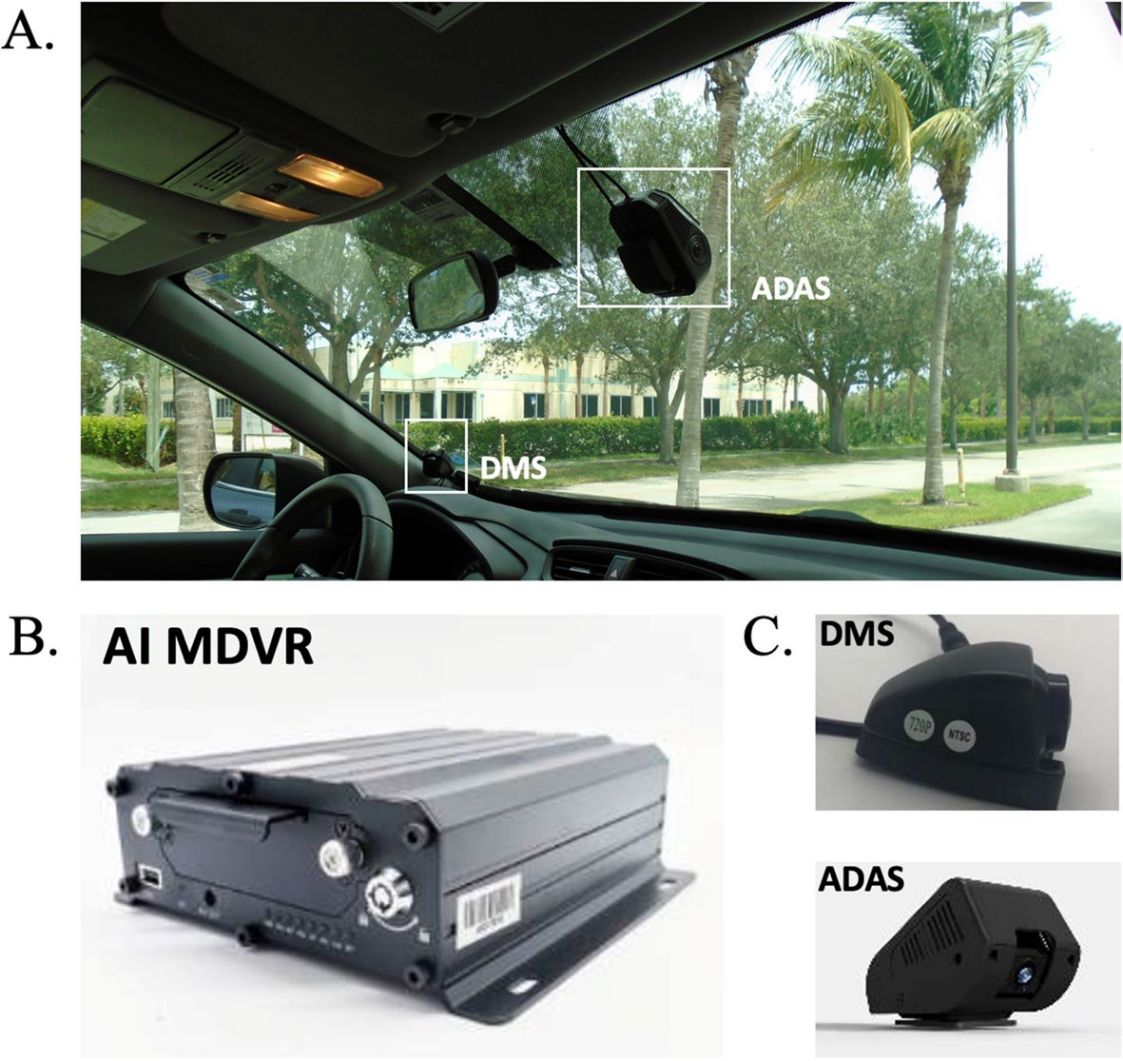
**A** Installation of AI (Artificial intelligence)-embedded cameras. The Advanced Driver Assistance System (ADAS) camera faces outwards and is located at the top center of the windshield. The Driver Monitoring System (DMS) faces the participant driver and is located at the bottom left of the windshield. **B** Close-up of the Artificial Intelligence Module Digital Video Recorder (MDVR). **C** The ADAS and the DMS

The video unit consists of the MDVR (Digital Video Recorder), a driver-facing camera, and a forward-facing camera (see Fig. 2). The MDVR has a storage unit with a capacity of 256 GB, which allows storing the video from both cameras for a period of 3 months. The MDVR has built-in AI functions that analyze video in real-time. The driver-facing camera is mounted in the left corner of the windshield and is directed to the driver’s face to analyze his/her behavior and facial expressions. Table 1 shows a list of indices that are analyzed by the driver- facing camera.

**Table 1:** Driver-facing camera indices

Behavior Indices	Description
Face detection	AI algorithm detects the face of the driver and the driver’s features.
Eye detection	AI algorithm detects the eyes and whether they are open or closed.
Yawning	Using eye and mouth detection, yawning is detected.
Distraction	Head-pose estimation technique is applied to detect distraction.
Smoking Mobile Phone Use	Driver’s smoking and use of mobile phones are detected using an AI algorithm.

The forward-facing camera is mounted under the rear-view mirror and is used to record events external to the vehicle. Table 2 shows the list of indices that are recorded and analyzed using the front-facing camera.

**Table 2:** Forward-facing camera indices

Behavior Indices	Description
Traffic Sign Detection	AI algorithm detects traffic signals and monitors if the driver runs a red traffic light.
Object Detection	AI algorithm detects objects on the road, such as pedestrians or cyclists crossing the road, curbs or barriers, and nearby vehicles.
Lane Crossing	AI algorithm detects lane departure.
Near-Collision	AI algorithm can detect an object or a vehicle that is close to the driver’s car.
Pedestrian Detection	AI algorithm detects whether the driver yields when a pedestrian crosses the street.

### Cognitive battery

#### Measure selection

The team assembled the neuropsychological assessment protocol with two aims: 1. To classify participants into three diagnostic groups: mild cognitive impairment (MCI), early dementia, and unimpaired (normal). 2. To detect subtle changes over time in these measures independent of clinical assessments, particularly in executive function and visual attention, which are the cognitive domains most strongly associated with driving variables.

To fulfill these aims, two groups of tests were assembled: A clinical battery including assessments of cognition, functioning in daily activities, and mood (depression), and an additional set of tests including executive function and attention.

### Clinical battery

#### Global cognitive function

*The Montreal Cognitive Assessment (MoCA)*, is a measure of global cognitive function [17] originally developed to detect mild cognitive impairment (MCI) and now frequently used as a screening test. It is a 10-min test that assesses short-term memory, visuospatial function, executive function, attention, concentration, working memory, language, and orientation.

#### Executive function

*Trail Making Test* [19–21] is predominantly a measure of divided attention and two components of executive function, cognitive response set maintenance and shifting. The primary outcome measure for each TMT is time in seconds. Additionally, a derived Trails B/Trails A ratio will be calculated to obtain a relatively independent measure of executive control [22].

*The Stroop-Color Word Test (SCWT)* is a neuropsychological test used in both clinical and experimental settings [23]. This test measures the ability to inhibit cognitive interference that may occur while processing two stimuli simultaneously. The processing of one stimulus (e.g. color) may interfere with the second stimulus (e.g. word) processing, causing a Stroop Effect, in which the participant may confuse one stimulus for the other. Participants will be tested for color blindness prior to administration of this test.

#### Semantic memory

##### Loewenstein-Acevedo Scales of Semantic Interference and Learning (LASSI-L)

Participants are tasked with remembering two lists with 15 words each including fruits, musical instruments, or clothing. The words are presented one at a time on cards and are read by the participant. Participants are made aware of the semantic categories. This is followed by a free recall trial and then cued recall trials for each of the 3 categories. List A is presented again, and an additional cued recall trial for each category is conducted. A second list (List B) is subsequently presented, followed by a free recall trial, then three cued recall trials (one per category). List B is presented for a second time, with another round of cued recall trials. Then the participant is asked to think back to List A followed by the free recall and cued recall trials of List A. Participants are allowed 60 s during free recall trials and 20 s for each semantic category during cued recall trials. After 20 min, there is a delayed free recall of both lists. Previous analyses of the LASSI-L have demonstrated its sensitivity in differentiating between MCI and cognitively normal participants [24]. Semantic interference tasks similar to the LASSI-L have been shown to be valid across participants with different cultural backgrounds [25].

##### Logical memory

The Craft Story is a logical memory test that typically takes 20 min to administer [26]. The examiner reads a short story of approximately 60 words aloud and then asks the individual to recall as much detail as possible both immediately and after a 20-min delay.

#### Visuospatial and visuomotor

*The Benson Figure Drawing (BFD)* [27] has been used to evaluate visuospatial cognition in dementia. The patient is asked to copy a figure with no limit on response time.

#### Language

##### Naming

The Multilingual Naming Test (MINT) assesses confrontation naming with 32 pictures of objects [28]. It detects naming deficits in patients with MCI or AD [29].

*Verbal Fluency (VF)* will be assessed using category (animals) [30] and phonemic (letters P and F) fluency [31]. Successful performance on word fluency tasks requires executive functions such as inhibiting words that do not conform to the rules of the task. Although often conceptualized as measuring executive functioning, recent analyses showed that language processing is the critical component [32].

#### Functional ability measures

*The Functional Activities Questionnaire (FAQ)* measures instrumental activities of daily living (IADLs) affected by changes in cognition, such as preparing balanced meals and managing personal finances [33, 34].

*Everyday Cognition (ECog)* [35] consists of one global everyday function scale and six subscales (Everyday Memory, Language, Visuospatial Abilities, Planning, Organization, and Divided Attention). The ECog has shown sensitivity to MCI [35, 36].

#### Mood

The Geriatric Depression Scale-Short form (GDS-15) [37, 38] will be used to measure the level of depression symptom severity.

#### Clinical rating

*The Clinical Dementia Rating Scale (CDRS)* [39] is a 5-point scale used to quantify the severity of cognitive impairment symptoms. Six domains of cognitive performance are measured: Memory, Orientation, Judgement and Problem-solving, Community Affairs, Home and Hobbies, and Personal Care. These domains are relevant to the diagnosis and severity of Alzheimer’s Disease and Dementia. The CDRS score is derived from information collected from an informant (study partner) interview as well as a participant interview.

### Additional measures

#### Executive function

##### ***Cogstate: Identification (IDN) task***

The Identification (IDN) [40] task is a simple choice reaction time paradigm that measures reaction time and decision-making.

##### ***Arrow Flanker Task (AFT)***

The Flanker Task [41, 42] is a neuropsychological task that measures inhibition using non-verbal stimuli such as arrows. It assesses the ability to suppress unrelated responses.

##### **Cogstate: One Back (ONB) and Two Back (TWOB) task**

These two tasks [40] assess working memory and attention.

*Cogstate: The Groton Maze Learning Test (GMLT)* [40] is designed to measure executive function using a maze learning paradigm.

#### Visual attention

*The Useful Field of View test (UFOV)* is a measure of three perceptual-cognitive abilities: processing speed, divided attention, and selective attention. These three tasks represent higher-order cognitive functioning required for safe vehicle driving [43]. The UFOV software (version 6.1.4; Visual Awareness Research Group Inc.) will be used.

### Clinical diagnosis

A clinical diagnosis will be made using an algorithm that has demonstrated reliability and validity in the diagnosis of MCI and dementia [44]. It combines the CDR clinical ratings with the neuropsychological test-based diagnosis (see Table 3). Cases in which there is a disagreement between the clinical ratings and test results will be presented to an expert panel of 4 clinical experts (2 neuropsychologists, a neurologist, and a geriatric nursing specialist) at a consensus diagnostic panel meeting.

**Table 3:** Algorithmic diagnosis including Clinical Dementia Rating (CDR) scores and neuropsychological test-based diagnosis of normal, amnestic and non-amnestic Mild Cognitive Impairment (aMCI; naMCI) and early (eMCI) and late MCI (LMCI)

CDR Sum of Boxes	Neuropsychological Diagnosis (NpDx)			
	Normal	aMCI	naMCI	Dementia
0	Normal	PreMCI –NP	PreMCI-NP	Consensus Conference
0.5–2.0	PreMCI Clinical	eMCI	eMCI	Consensus Conference
2.5–4.0	PreMCI Clinical	eMCI	eMCI	LMCI
4.5+	Consensus Conference	Dementia	Dementia	Dementia

[Rule 1: CDR-sb score > 4.0 indicates dementia diagnosis (except when NpDx is Normal)]

[Rule 2: CDR-sb score of 2.5 to 4.0 indicates an LMCI diagnosis (except when NpDx is Normal)]

[Rule 3: CDR-sb score of 0.5 to 2.0 indicates an eMCI diagnosis (except when NpDx is Normal)]

[Rule 4: Pre-MCI is diagnosed when CDR-sb 0.5–4.0 and NpDx is normal, or CDR-sb = 0 and NpDx = aMCI or naMCI]

*CDR-sb* = CDR sum of boxes, *aMCI* = Amnestic MCI, *naMCI* = Non-amnestic MCI, *LMCI* = Late MCI, *eMCI* = Early MCI

### Study procedures

#### Testing locations

Participants will be recruited from Broward and Palm Beach Counties in Southeast Florida. Florida Atlantic University (FAU) has several campuses in Broward and Palm Beach Counties. FAU’s Memory and Wellness Center is on the main Boca Raton campus and will serve as a testing and sensor installation/maintenance site as will the Clinical Research Unit, also on the main campus. Secondary sites will be on the Davie campus in central Broward, and several cooperating places of worship and community centers for the convenience of those older drivers who customarily drive within a restricted range. A subaward to colleagues in Psychology, Engineering, and Nursing at the University of Central Florida (UCF) will support the recruitment and testing of eligible participants on the UCF campus in Orlando, Florida. Data will be transmitted securely to FAU for analysis.

#### Enrollment procedures

Older drivers who express an interest in participation will receive an explanation of the study and the activities that participation entails. They will then be screened for eligibility and proceed to the first set of assessments. An installation team will place the telematics and video sensors in participants’ vehicles while the cognitive tests are administered.

#### Scheduling over 3 years

The project aims to identify cognitive changes over time that may be associated with indicators from the in-vehicle sensors. Therefore, cognitive assessments are administered every 3, 6, and 12 months (depending on the tests) for 3 years, for a total of 13 quarterly assessments. Every 3 months the following cognitive assessments will be administered: MoCA, FAQ, GDS-15, and the DSAQ (Driver Sensor Acceptability Survey which responds to Aim 3 of the study (See list of abbreviations)). The IDN, GMLT, ONB, and TWOB will be administered at every time point except for Visit 1 in order to keep the first visit under 3 h. Since we will use parallel forms of these tests, they do not need a double baseline. The following assessment tools will be administered beginning with Visit 2 and again on Visit 3 for the double baseline, then every 6 months: ECog, BFD, TMT, AFT, SCWT, VF, and MINT. The UFOV will be administered on Visits 1 and 2, then repeated every 6 months. Lastly, the LASSI-L and Craft Story will be administered at visits 1 and 2 for the double baseline and then every 12 months. The MMSE will be administered on visits 1, 5, 9, and 13.

### Preparation of assessors and psychometricians

Experienced nursing and psychology students and postdoctoral fellows will perform the clinical and cognitive assessments. These research assistants will go through intensive training in the administration and scoring of the assessments. They are first exposed to these procedures via training videos followed by one-to-one observation sessions with clinicians and experienced psychometricians who explain the administration and scoring procedures for each assessment measure in detail. After training, each research assistant will submit videos of themselves performing the clinical and psychometric assessments with non-participant volunteers. Individual feedback is provided regarding any deviations from the standard administration, and if necessary, a second video is requested. Approval to conduct assessments independently is obtained after completing an additional 4–6 shadowing sessions, during which the newly trained research assistants will be observed administering the assessments.

The clinical diagnosis will be made by a team of neurologists, neuropsychologists, and geriatric nurses who review the clinical and psychometric data using the Algorithmic Diagnosis procedure described above. Clinical personnel are blinded to the data driving the decision in order to avoid potential bias in the diagnosis.

#### Controlling for practice effects

Important issues in this study of cognitive change are the potential for practice and retest effects in performance on repeatedly administered cognitive tests, a problem common to longitudinal studies. Although we may assume that these effects are observed in all clinical groups, they may not be equivalent across groups.

While there is no clear consensus on the best methods to address this problem, we will address it by:

- Using alternative forms with equivalent psychometric properties where available. The IDN, ONB, TWOB, and the GMLT are short and repeatable assessments that are unaffected by language, therefore we are able to have a large stimulus set that is controlled and randomized so that each participant has a unique stimulus set at each time point. The AFT uses a set of pseudo randomizations controlling for the number of congruent and incongruent trials.
- The most prominent practice effects generally take place between the first and the second administration of a test. For this reason, we will use a double baseline design in which the second assessment (3 months after the first) will be used as the baseline for all cognitive measures.
- In the statistical analysis we will include reliable change indices (RCIs), and regression base analyses with correction for practice effects.

### Statistical analysis

#### Data processing phase

Data processing will occur in three steps. First, several preliminary models will be created to provide greater flexibility in analyzing the relationships between the driving behaviors and cognition over time. Various models such as linear mixed models and non-linear functions, such as splines, will be assessed.

The second processing step is feature extraction. Variables will be created from the raw data, with mean, standard deviation, and assessment of normality analyzed. During this step the telematic and video data will be standardized so that all of the features will account for driving speed and length of time driving during each trip. Since the telematic and video data have varying scales, data will be normalized using the minmax approach. Lastly, external weather and traffic conditions data will be added to the database.

The third processing step is the reduction of the number of features to prevent overfitting the model as well as to reduce the complexity of the model. Two strategies will be used to complete the feature selection step. Elastic net regression adds a penalty and bias to the models resulting in the retention of only the critical variables. These results will be compared to the results for the second method, SHAP (Shapley Additive exPlanations) analysis, which is a global feature selection method that will be used to compute the feature importance of each variable in predicting cognitive change [45].

#### Predictive modeling phase

Two different methods will be used to model the prediction of changes in cognitive functioning followed by a sensitivity analysis. First, a traditional predictive modeling method using a linear mixed effects (LME) model with time varying covariates will be conducted [46]. This LME method will use the processed data aggregated at weekly, monthly, and 3-month intervals along with the features determined by the SHAP analysis. The second method will use a machine learning recurrent neural network (RNN) approach to loop over and pass information from one step to another in the network allowing for the capability to effectively incorporate temporal (time) dependencies in longitudinal data [47]. With RNN, data contains data sequences from k time steps at each time point (ti), input features (Xti), and internal state (memory) from the previous time step (ht(i−1)) allowing for RNN to identify patterns hidden in the sequence of the data not detectible by conventional neural networks [48]. The Driving Behavior Indices (DBIs) algorithms generated will be assessed for ability to predict changes in overall cognitive functioning as well as other specific domains.

#### Driving behavior (normalized driver behavior indices)

Driver Behavior Indices (DBIs) will be estimated from the telematics and vision sensor data. The selection of the DBIs is designed to reflect older drivers’ cognitive function and driving performance. To account for the variation in participants’ driving frequency and mileage, the DBIs will be normalized by the total number of trips, left turns, traveled intersections and/or total mileages. The DBIs will be evaluated for each driver and will be summarized on a daily, weekly, and monthly basis. DBIs are classified into four categories. Examples of DBIs are shown in Table 4.

**Table 4:** Driver behavior indices

**Categories**	**DBIs**	**Data Analytics**
**Travel Patterns**	number of trips, miles driven, miles on the highway, miles during the night, daytime, and severe weather, highway miles, etc.	map-matching, data queries, map data, weather data
**Abnormal Driving**	wayfinding, getting lost, ignoring traffic signals and signs, near-collision events, distraction, drowsiness, etc.	machine vision, shortest path, outlier detection, trajectory clustering, frequent graph mining
**Reaction time**	reaction time to traffic light change, front-vehicle taillight, pothole, etc.	vision sensing, data fusion, vibration analysis, machine learning
**Braking Patterns**	eye movements and IMU data at stop signs, traffic signals, tail lights, losing focus, potholes, etc.	signal processing, gaze estimation, data mining, machine vision

#### Travel patterns

DBIs include travel patterns that will be analyzed based on vehicles’ trajectories, combined with map-matching algorithms to relate high-precision positioning data to map data (e.g., traveled road names, the types of roads, and speed limits). The inclusion of map-matching algorithms and weather data is required to analyze trips on freeways and during severe weather conditions, respectively. These travel-pattern-related DBIs are known to be indicative of the changes in older drivers’ cognition and physical functions since they tend to incorporate deliberate avoidance strategies to compensate for age-related deficits [49].

#### Abnormal driving

Advancing abnormal driving pattern detection requires novel multi-attributed spatial network queries for Spatial Network Database (SNDB) due to the heterogeneity of data sources, such as map data, weather, and location of stop signs. For example, queries for identifying abnormal driving patterns need to combine interrelated risk factors under the unified index and analyze these factors based on topological and geospatial reference systems. Furthermore, drivers’ potential route selection can be affected by traffic volume and weather conditions, which also necessitate developing domain specific SNDB queries to achieve an accurate estimation of abnormal traffic patterns. The identification of ignoring traffic signals or stop signs will use the temporally detailed network status (e.g., volume and speed), the number of lanes, and localized spatial information.

#### Reaction time

The reaction time estimation requires the integration of driving scene awareness, eye tracking, and telematics data. The reaction time to traffic lights and taillights will be analyzed by considering various factors, such as distance to the objects and vehicle speed.

#### Braking patterns

Hard-braking-related DBIs will be used to monitor older drivers’ braking patterns and eye movements. Highly-detailed braking-related data will be collected from our telematics and vision sensors. For example, the data fusion of high-precision trajectories, map data, and machine vision will indicate the causes of hard braking events (e.g., stop-and-go conditions at an intersection, tailgating, losing focus, road conditions). Vision sensors will relate hard braking to stop signs or traffic lights, providing detailed glance patterns at an intersection. Importantly, the data analytics of braking patterns must consider whether drivers might be aware of stop signs, traffic signals, and potholes in advance. This can be closely related to memory function. For example, drivers are usually aware of the location of potholes and stop signs in advance when they repeatedly drive the routes in their daily life (e.g., commuting, grocery shopping). If there are changes in their cognitive functioning, they would react differently to traffic signs or potholes than they did prior to the changes.

To address the third study aim, acceptability of the installed sensor systems, the psychometric properties of the DSAQ will be evaluated and differential effects by demographic group (age, gender, ethnic group membership, education) evaluated. Thematic analysis [50] of the interview data on acceptability will provide explanations for the ratings obtained on the DSAQ.

## Ethical considerations

Prior to enrollment, all potential participants receive an explanation of the study including what participation entails and sign a consent approved by the Florida Atlantic University Committee for the Protection of Human Subjects. Study procedures and consent processes were reviewed and approved by the University Committee for the Protection of Human Subjects.

## Anticipated challenges

Several challenges to the successful implementation of this protocol are anticipated. Participation in the study requires a commitment from the participant to attend testing and data retrieval from the installed sensors four times a year for 3 years. A secondary concern is possible. participant hesitation to allow a video camera to focus on the participant’s face. Should this become a problem in the recruitment of participants, otherwise eligible enrollees will be allowed to forego the driver-facing camera, and a comparison of the accuracy of cognitive change detection with and without the driver-facing video data will be added to the data analysis plan.

There may also be some concern about driver information becoming available to insurers. The Certificate of Confidentiality issued by the National Institutes of Health prevents insurance companies from accessing this information without participant consent and should alleviate this concern.

A more technical concern is the ability to assign driving data to the correct individual when a vehicle is driven by more than one person, most likely spouses who share the vehicle. The solution will be to develop and train a facial comparison algorithm to distinguish the two drivers.

## Discussion

A naturalistic 3-year longitudinal study will be conducted to test the ability of an in-vehicle sensor system of telematic and video sensors to obtain data that can signal a change in the driver’s cognitive status will be tested. The innovation of this research project lies in the unobtrusive, rapidly, and readily available in-vehicle sensing and monitoring system built upon modern open-source hardware and software using existing techniques to develop and customize the components and configure them for this new purpose. We have hypothesized that this system will be capable of detecting a change in cognitive status among older drivers who are developing MCI or exhibiting symptoms of early-stage dementia.

## Conclusions

It is well established that individuals with progressive dementias are eventually unable to drive safely, yet many remain unaware of their cognitive decrements. Current screening and evaluation services can test only a small number of individuals with cognitive concerns, missing many who need to know if they require treatment. Given the increasing number of sensors being installed in passenger vehicles and their increasing acceptability, reconfigured in-vehicle sensing systems could provide widespread, low-cost early warnings of cognitive change and decline to the large number of older drivers on the road in the U.S. and elsewhere. The proposed testing and evaluation of a readily and rapidly available, unobtrusive in-vehicle sensing system could provide the first step toward future widespread, low-cost, early warnings of cognitive change for this large number of older drivers.

## Data Availability

Prior to completion of the study, we will apply to an NIH-supported scientific data repository related to AD/ADRD to provide access to deidentified study data. This will not include live video data but will include the data on driver behavior generated from the videos. The dataset referenced in the current report will be available in de-identified form from the corresponding author on reasonable request.

## Abbreviations

4G: A mobile communications standard allowing wireless internet access at high speed
AD: Alzheimer’s Disease
AFT: Arrow Flanker Task
AI: Artificial Intelligence
aMCI: Amnestic Mild Cognitive Impairment
ARIMA: Auto Regressive Integrated Moving Average This is a statistical analysis model that uses time series data to either better understand the data set or to predict future trends
BFD: Benson Figure Drawing
CAS-IRT: Cognitive Abilities Screening Instrument scored using Item Response Theory
CDR: Clinical Dementia Rating
CDR-sb: Clinical Dementia Rating sum of boxes
COG: Course Over Ground
DBI: Driving Behavior Indices
DSAQ: Driver Sensor Acceptability Survey
ECog: Everyday Cognition
FAU: Florida Atlantic University
FAQ: Functional Activities Questionnaire
GDS-15: Geriatric Depression Scale-Short form
G-force: Gravitational Force
GMLT: Groton Maze Learning Test
GPS: Global positioning System
Hz: Hertz
IDN: Identification
IMU: Inertial Measurement Unit
LASSI-L: Loewenstein-Acevedo Scales of Semantic Interference and Learning
mA: Milliamp
LMCI: Late Mild Cognitive Impairment
MCI: Mild Cognitive Impairment
MDVR: Module Digital Video Recorder
MINT: Multilingual Naming Test
MMSE: Mini-Mental State Examination
MoCA: Montreal Cognitive Assessment
MWC: Memory and Wellness Center
naMCI: Non-amnestic MCI
NpDx: Neuropsychological Diagnosis
OBD: On-board diagnostics
ONB: One Back Tasks test
Pre-MCI: Pre-Mild Cognitive Impairment
RCI: Reliable Change Indices
SCWT: Stroop-Color Word Test
TMT: Trail Making Test
TWOB: Two Back Task test
UCF: University of Central Florida
UFOV: Useful Field of View test
U.S.: United States
VF: Verbal Fluency

## References

[1] Rajan KB, Weuve J, Barnes LL, McAninch EA, Wilson RS, Evans DA. Population estimate of people with clinical Alzheimer’s disease and mild cognitive impairment in the United States (2020–2060). U.S. National Library of Medicine. Available from: https://pubmed.ncbi.nlm.nih.gov/34043283/. Cited 2023 Sept 6. 10.1002/alz.12362 PMC9013315 34043283

[2] Aita SL, Beach JD, Taylor SE, Borgogna NC, Harrell MN, Hill BD. Alzheimer’s disease facts and figures. 2022. Available from: https://www.alz.org/alzheimers-dementia/facts-figures. Cited 2023 Sept 6.

[3] Ott BR, Jones RN, Noto RB, Yoo DC, Snyder PJ, Bernier JN, et al. Brain amyloid in preclinical Alzheimer’s disease is associated with increased driving risk. U.S. National Library of Medicine; 2016. Available from: https://www.ncbi.nlm.nih.gov/pmc/articles/PMC5318288/. Cited 2023 Sept 6.

[4] Meuleners LB, Stevenson M, Chow K, Ng J. Motor vehicle crashes and dementia: a population-based study. U.S. National Library of Medicine; 2016. Available from: https://pubmed.ncbi.nlm.nih.gov/27171906/. Cited 2023 Sept 6.

[5] Chee JN, Rapoport MJ, Molnar F, Herrmann N, O’Neill D, Marottoli R, Mitchell S, Tant M, Dow J, Ayotte D, Lanctôt KL, McFadden R, Taylor JP, Donaghy PC, Olsen K, Classen S, Elzohairy Y, Carr DB. Update on the risk of motor vehicle collision or driving impairment with dementia: a collaborative international systematic review and meta-analysis. U.S. National Library of Medicine; 2017. Available from: https://pubmed.ncbi.nlm.nih.gov/28917504/. Cited 2023 Sept 6. 10.1016/j.jagp.2017.05.007 28917504

[6] Fraade-Blanar LA, Ebel BE, Larson EB, Sears JM, Thompson HJ, Chan KCG, Crane PK. Cognitive decline and older driver crash risk. U.S. National Library of Medicine; 2016. Available from: https://pubmed.ncbi.nlm.nih.gov/29667168/. Cited 2023 Sept 6. 10.1111/jgs.15378 PMC6541224 29667168

[7] Barco PP, Baum CM, Ott BR, Ice S, Johnson A, Wallendorf M, Carr DB. Driving errors in persons with dementia. U.S. National Library of Medicine; 2015. Available from: https://pubmed.ncbi.nlm.nih.gov/26140521/. Cited 2023 Sept 6.

[8] Pavlou D, Papadimitriou E, Antoniou C, Papantoniou P, Yannis G, Golias J, et al. Driving behavior of drivers with mild cognitive impairment and Alzheimer’s disease: a driving simulator study. 2015. Available from: https://www.researchgate.net/publication/282185188_Driving_behaviour_of_drivers_with_Mild_Cognitive_Impairment_and_Alzheimer’s_Disease_A_Driving_Simulator_Study. Cited 2023 Sept 6.

[9] Eby DW, Adler G, LeBlanc D, Molnar LJ, Silverstein NM. Driving behaviors in early stage dementia: a study using in-vehicle technology. U.S. National Library of Medicine; 2012. Available from: https://pubmed.ncbi.nlm.nih.gov/23036413/. Cited 2023 Sept 6. 10.1016/j.aap.2011.11.021 23036413

[10] Seelye A, Mattek N, Sharma N, Witter P, Brenner A, Wild K, Dodge H, Kaye J. Passive assessment of routine driving with unobtrusive sensors: a new approach for identifying and monitoring functional level in normal aging and mild cognitive impairment. U.S. National Library of Medicine; 2017. Available from: https://pubmed.ncbi.nlm.nih.gov/28731434/. Cited 2023 Sept 6. 10.3233/JAD-170116 PMC5640318 28731434

[11] Di X, Shi R, DiGuiseppi C, Eby DW, Hill LL, Mielenz TJ, Molnar LJ, Strogatz D, Andrews HF, Goldberg TE, Lang BH, Kim M, Li G. Using naturalistic driving data to predict mild cognitive impairment and dementia: preliminary findings from the longitudinal research on Aging Drivers (LongROAD) study. U.S. National Library of Medicine; 2021. Available from: https://pubmed.ncbi.nlm.nih.gov/33922735/. Cited 2023 Sept 6. 10.3390/geriatrics6020045 PMC8167558 33922735

[12] Bayat S, Babulal GM, Schindler SE, Fagan AM, Morris JC, Mihailidis A, et al. Identifying preclinical Alzheimer disease from driving patterns: a machine learning approach. 2012. Available from: https://alz-journals.onlinelibrary.wiley.com/doi/10.1002/alz.057316. Cited 2023 Sept 6.

[13] Davis JD, Babulal GM, Papandonatos GD, Burke EM, Rosnick CB, Ott BR, Roe CM. Evaluation of naturalistic driving behavior using in-vehicle monitoring technology in preclinical and early Alzheimer’s disease. U.S. National Library of Medicine; 2020. Available from: https://pubmed.ncbi.nlm.nih.gov/33192943/. Cited 2023 Sept 6. 10.3389/fpsyg.2020.596257 PMC7653196 33192943

[14] Huisingh C, Owsley C, Wadley VG, Levitan EB, Irvin MR, MacLennan P, McGwin G. General cognitive impairment as a risk factor for motor vehicle collision involvement: a prospective population-based study. U.S. National Library of Medicine; 2018. Available from: https://pubmed.ncbi.nlm.nih.gov/29600251/. Cited 2023 Sept 6. 10.3390/geriatrics3010011 PMC5869692 29600251

[15] Faul F, Erdfelder E, Buchner A, Lang A-G. Statistical Power analyses using G*Power 3.1: tests for correlation and regression analyses - behavior research methods. Springer-Verlag; 2009. Available from: https://link.springer.com/article/10.3758/BRM.41.4.1149. Cited 2023 Sept 6. 10.3758/BRM.41.4.1149 19897823

[16] Han M, Lee E. Effectiveness of mobile health application use to improve health behavior changes: a systematic review of randomized controlled trials. U.S. National Library of Medicine; 2018. Available from: https://pubmed.ncbi.nlm.nih.gov/30109154/. Cited 2023 Sept 6.

[17] Nasreddine ZS, Phillips NA, Bédirian V, Charbonneau S, Whitehead V, Collin I, Cummings JL, Chertkow H. The Montreal Cognitive Assessment, MOCA: a brief screening tool for mild cognitive impairment. U.S. National Library of Medicine; 2005. Available from: https://pubmed.ncbi.nlm.nih.gov/15817019/. Cited 2023 Sept 6. 10.1111/j.1532-5415.2005.53221.x 15817019

[18] Birdies. Factors that affect GPS accuracy. Available from: https://help.18birdies.com/article/537-factors-that-affect-gps-accuracy. Cited 2023 Sept 7.

[19] Reitan RM. The relation of the trail making test to organic brain damage. U.S. National Library of Medicine; 1955. Available from: https://pubmed.ncbi.nlm.nih.gov/13263471/. Cited 2023 Sept 7.

[20] Arbuthnott K, Frank J. Trail making test, part B as a measure of executive control: Validation using a set-switching paradigm. U.S. National Library of Medicine; 2000. Available from: https://pubmed.ncbi.nlm.nih.gov/10923061/. Cited 2023 Sept 7. 10.1076/1380-3395(200008)22:4;1-0;FT518 10923061

[21] Dobbs BM, Shergill SS. How effective is the trail making test (parts A and B) in identifying cognitively impaired drivers? U.S. National Library of Medicine; 2013. Available from: https://pubmed.ncbi.nlm.nih.gov/23896609/. Cited 2023 Sept 7. 10.1093/ageing/aft073 23896609

[22] Drane DL, Yuspeh RL, Huthwaite JS, Klingler LK. Demographic characteristics and normative observations for derived-trail making test indices. U.S. National Library of Medicine; 2002. Available from: https://pubmed.ncbi.nlm.nih.gov/11877550/. Cited 2023 Sept 7. 11877550

[23] Golden JC, Freshwater SM. 2002. Available from: https://www.worldcat.org/title/Stroop-color-and-word-test-:-a-manual-for-clinical-and-experimental-uses/oclc/811788223. Cited 2023 Sept 7.

[24] Loewenstein DA, Curiel RE, Greig MT, Bauer RM, Rosado M, Bowers D, Wicklund M, Crocco E, Pontecorvo M, Joshi AD, Rodriguez R, Barker WW, Hidalgo J, Duara R. A novel cognitive stress test for the detection of preclinical Alzheimer disease: discriminative properties and relation to amyloid load. U.S. National Library of Medicine; 2016. Available from: https://pubmed.ncbi.nlm.nih.gov/27160985/. Cited 2023 Sept 7. 10.1016/j.jagp.2016.02.056 PMC5026876 27160985

[25] Rosselli M, Tappen RM, Newman D. Semantic interference test: Evidence for culture and education fairness from an ethnically diverse sample in the USA. U.S. National Library of Medicine; 2019. Available from: https://pubmed.ncbi.nlm.nih.gov/29688251/. Cited 2023 Sept 7. 10.1093/arclin/acy037 29688251

[26] Craft S, Newcomer J, Kanne S, Dagogo-Jack S, Cryer P, Sheline Y, et al. Memory improvement following induced hyperinsulinemia in Alzheimer’s disease. Neurobiol Aging. 1996. Available from: https://www.sciencedirect.com/science/article/pii/0197458095020020. Cited 2023 Sept 7. 10.1016/0197-4580(95)02002-0 8786794

[27] Possin KL, Laluz VR, Alcantar OZ, Miller BL, Kramer JH. Distinct neuroanatomical substrates and cognitive mechanisms of figure copy performance in Alzheimer’s disease and behavioral variant frontotemporal dementia. U.S. National Library of Medicine; 2011. Available from: https://pubmed.ncbi.nlm.nih.gov/21029744/. Cited 2023 Sept 7. 10.1016/j.neuropsychologia.2010.10.026 PMC3005024 21029744

[28] Gollan TH, Weissberger GH, Runnqvist E, Montoya RI, Cera CM. Self-ratings of spoken language dominance: a multi-lingual naming test (MINT) and preliminary norms for young and aging Spanish-English bilinguals. U.S. National Library of Medicine; 2012. Available from: https://pubmed.ncbi.nlm.nih.gov/25364296/. Cited 2023 Sept 7. 10.1017/S1366728911000332 PMC4212892 25364296

[29] Stasenko A, Jacobs DM, Salmon DP, Gollan TH. The multilingual naming test (MINT) as a measure of picture naming ability in Alzheimer’s disease. U.S. National Library of Medicine; 2019. Available from: https://pubmed.ncbi.nlm.nih.gov/31248465/. Cited 2023 Sept 7. 10.1017/S1355617719000560 PMC6757330 31248465

[30] Benton AL. Development of a multilingual aphasia battery. progress and problems. U.S. National Library of Medicine; 1969. Available from: https://pubmed.ncbi.nlm.nih.gov/5820858/. Cited 2023 Sept 7. 10.1016/0022-510x(69)90057-4 5820858

[31] Benton AL, Hamsher DS, Sivan AB. Contributions to Neuropsychological Assessment. A Clinical Manual (2nd ed.). New York: Oxford University Press; 1994.

[32] Whiteside DM, Kealey T, Semla M, Luu H, Rice L, Basso MR, Roper B. Verbal fluency: Language or executive function measure? U.S. National Library of Medicine; 2016. Available from: https://pubmed.ncbi.nlm.nih.gov/26111011/. Cited 2023 Sept 7. 10.1080/23279095.2015.1004574 26111011

[33] Pfeffer RI, Kurosaki TT, Harrah CH, Chance JM, Filos S. Measurement of functional activities in older adults in the community. U.S. National Library of Medicine; 1982. Available from: https://pubmed.ncbi.nlm.nih.gov/7069156/. Cited 2023 Sept 7.

[34] Tappen RM, Rosselli M, Engstrom G. Evaluation of the functional activities questionnaire (FAQ) in Cognitive Screening across four American ethnic groups. U.S. National Library of Medicine; 2010. Available from: https://pubmed.ncbi.nlm.nih.gov/20473827/. Cited 2023 Sept 7. 10.1080/13854040903482855 20473827

[35] Farias ST, Mungas D, Reed BR, Harvey D, Cahn-Weiner D, Decarli C. MCI is associated with deficits in everyday functioning. U.S. National Library of Medicine; 2006. Available from: https://pubmed.ncbi.nlm.nih.gov/17132965/. Cited 2023 Sept 7. 10.1097/01.wad.0000213849.51495.d9 PMC2880610 17132965

[36] van Harten AC, Mielke MM, Swenson-Dravis DM, Hagen CE, Edwards KK, Roberts RO, Geda YE, Knopman DS, Petersen RC. Subjective cognitive decline and risk of MCI: the Mayo Clinic Study of Aging. U.S. National Library of Medicine; 2018. Available from: https://pubmed.ncbi.nlm.nih.gov/29959257/. Cited 2023 Sept 7. 10.1212/WNL.0000000000005863 PMC6070384 29959257

[37] Yesavage JA, Brink TL, Rose TL, Lum O, Huang V, Adey M, Leirer VO. Development and validation of a geriatric depression screening scale: a preliminary report. U.S. National Library of Medicine; 1982. Available from: https://pubmed.ncbi.nlm.nih.gov/7183759/. Cited 2023 Sept 7. 10.1016/0022-3956(82)90033-4 7183759

[38] Sheikh JL, Yesavage JA. Geriatric Depression Scale (GDS): recent evidence and development of a shorter version. American Psychological Association; 1986. Available from: https://psycnet.apa.org/record/1988-34658-001. Cited 2023 Sept 7.

[39] Morris JC. The clinical dementia rating (CDR): current version and scoring rules. U.S. National Library of Medicine; 1993. Available from: https://pubmed.ncbi.nlm.nih.gov/8232972/. Cited 2023 Sept 7. 10.1212/wnl.43.11.2412-a 8232972

[40] CogState Company Ltd (2009). CogState TM.

[41] Millisecond Software. Inquisit 6 Arrow Flanker Task [Computer software]. 2021. Retrieved from https://www.millisecond.com.

[42] Ridderinkhof KR, van der Molen MW, Band GP, Bashore TR. Sources of interference from irrelevant information: a developmental study. U.S. National Library of Medicine; 1997. Available from: https://pubmed.ncbi.nlm.nih.gov/9178963/. Cited 2023 Sept 7. 10.1006/jecp.1997.2367 9178963

[43] Edwards JD, Vance DE, Wadley VG, Cissell GM, Roenker DL, Ball KK. Reliability and validity of useful field of view test scores as administered by Personal Computer. U.S. National Library of Medicine; 2005. Available from: https://pubmed.ncbi.nlm.nih.gov/16019630/. Cited 2023 Sept 7. 10.1080/13803390490515432 16019630

[44] Duara R, Loewenstein DA, Greig M, Acevedo A, Potter E, Appel J, Raj A, Schinka J, Schofield E, Barker W, Wu Y, Potter H. Reliability and validity of an algorithm for the diagnosis of normal cognition, mild cognitive impairment, and dementia: implications for multicenter research studies. U.S. National Library of Medicine; 2010. Available from: https://pubmed.ncbi.nlm.nih.gov/20306566/. Cited 2023 Sept 7. 10.1097/jgp.0b013e3181c534a0 PMC2844658 20306566

[45] Plate JDJ, van de Leur RR, Leenen LPH, Hietbrink F, Peelen LM, Eijkemans MJC. Incorporating repeated measurements into prediction models in the Critical Care Setting: a Framework, systematic review and meta-analysis. U.S. National Library of Medicine; 2019. Available from: https://pubmed.ncbi.nlm.nih.gov/31655567/. Cited 2023 Sept 7. 10.1186/s12874-019-0847-0 PMC6815391 31655567

[46] Newman D, Newman I, Hitchcock JH. Cognitive analytics: concepts, methodologies, tools, and applications. 2020.

[47] Tabarestani S, Aghili M, Shojaie M, Freytes C, Cabrerizo M, Barreto A, et al. 2019. Available from: https://ieeexplore.ieee.org/document/8834556. Cited 2023 Sept 7.

[48] Goodfellow I, Bengio Y, Courville A. Deep learning. 2016.

[49] De Raedt, Ponjaert-Kristoffersen. The relationship between cognitive/neuropsychological factors and car driving performance in older adults. 2000. Available from: https://psycnet.apa.org/record/2000-12632-014. 10.1111/j.1532-5415.2000.tb03880.x 11129759

[50] Hsieh HF, Shannon SE (2005). Three approaches to qualitative content analysis. Qual Health Res.

